# Editorial: Exploring impacts of combined exposures to particles and chemicals on immune reactions across living organisms

**DOI:** 10.3389/ftox.2023.1148374

**Published:** 2023-03-06

**Authors:** Diana Boraschi, Albert Duschl, Iseult Lynch, Tobias Stoeger

**Affiliations:** ^1^ Shenzhen Institute of Advanced Technology (SIAT), Chinese Academy of Sciences (CAS), Shenzhen, China; ^2^ Institute of Biochemistry and Cell Biology (IBBC), National Research Council (CNR), Naples, Italy; ^3^ Stazione Zoologica Anton Dohrn (SZN), Napoli, Italy; ^4^ China-Italy Joint Laboratory of Pharmacobiotechnology for Medical Immunomodulation (SIAT CNR SZN), Shenzhen, China; ^5^ Department of Biosciences and Medical Biology, Allergy Cancer BioNano Research Center (ACBN), Paris-Lodron Universitaet Salzburg, Salzburg, Austria; ^6^ School of Geography, Earth and Environmental Sciences, University of Birmingham, Birmingham, United Kingdom; ^7^ Institute of Lung Health and Immunity (LHI), Comprehensive Pneumology Center Munich (CPC-M), Helmholtz Center Munich, and Member of the German Center of Lung Research (DZL) CPC-M, Munich, Germany

**Keywords:** immunotoxicity, nanoparticles, microplastics, microorganisms, chemicals

## 1 Editorial

The immune system of all living organisms has developed in order to preserve the integrity and functionality of the organism in the face of external invaders such as viruses, bacteria or pollutants. This encompasses a homeostatic role of patrolling and controlling the body tissues and organs, and a defensive role to face an external environment filled with microorganisms, particles and molecules of different nature, block the entry of potential threats and adapt to changes in the environment (Murphy et al., 2022).

### 1.1 History of exposure

Pollutants of different origins and nature can have an impact on immunity (e.g., Glencross et al., 2020). Alterations in immune responses caused by air and water pollutants can hamper human and environmental health, both in terms of inadequate reactivity (immunosuppression, increased susceptibility to infections and diseases) and in terms of excessive response (pathological inflammation, allergies, autoimmunity) (e.g., Reinmuth-Selzle et al., 2017). The recent concept of the exposome, the measure of all exposures experienced by individuals through their lifetime (Rappaport, 2011), fully applies to the interaction between pollutants and immunity, since the immune system has a memory (in vertebrates both innate and adaptive immune memory develop), which biases immune reactions to new challenges. Notably, the effect on immunity of the individual history of exposure (immunobiography) is a leading concept in the efforts of predicting possible inadequacy in immune reactivity to future challenges (Franceschi et al., 2017). Abundant cross-sectional and some longitudinal information is available on the immune effects caused by a number of individual pollutants, based mainly on experimental *in vitro* and *in vivo* models, in which animals or cells are exposed to the polluting agents (Joubert et al., 2020). However, much less is known regarding the more realistic circumstance of exposure to a combination of contaminants, in which we expect the combined exposure to have effects substantially different from the sum of individual pollutant’s effects. Studies on mixtures usually focus on a group of related substances (Holmstrup et al., 2010; Orr et al., 2020; Fair et al., 2021; Tooker et al., 2021), while the environment provides co-exposure to many substance pollutant classes at the same time (Soares et al., 2022).

### 1.2 Combined exposure to nanomaterials and other agents

In the case of engineered nanoparticles and nano/microplastics, carryover effects (i.e., effects arising from traces of chemicals used in previous synthesis runs) are common both in the case of chemicals used for particle synthesis, as for instance during additive manufacturing (Alijagic et al.), and from plasticizes added to plastic products (Hirt and Body-Malapel., 2020). Particles released into the environment can come into contact with different biotic and abiotic agents and adsorb them on their surface, giving rise to hybrid entities whose interaction with living organisms will depend on the new characteristics of the hybrid particles (Wheeler et al., 2021). A relevant example of hybrid entities are combustion-derived nanoparticles, e.g., soot and diesel exhaust particles, consisting of a nanosized carbon core with surface-adsorbed chemicals such as polycyclic aromatic hydrocarbon. The surface reactivity of the carbon particle can be modified by bioactive compounds, which might develop their toxicity only after cellular uptake by enzyme-mediated biotransformation (Stoeger et al., 2009). Thus, nanoparticles in the environment can have different impacts on the immune responses of all living organisms, following an interaction biased by the presence of an acquired biomolecule coating (Saptarshi et al., 2013; Boraschi et al., 2020; Pinsino et al., 2020; Wheeler et al., 2021), which can result in pathological reactions (Radauer-Preiml et al., 2016). Similarly, MPs can adsorb chemicals, getting coated with proteins and other biomolecules (biocorona) and interact with bacteria and viruses in the environment and within the organism, and consequently induce immune reactions that pristine microplastics cannot provoke (Yang et al., 2022a; Yang et al.). Notably, the type of particle makes a difference in the immune reaction elicited by the adsorbed agent. As an example, adsorption of SARS-CoV-2 on MPs does not change the viral structure or capacity to interact with target cells and, by promoting its transport, binding to MPs leads to enhanced infection of ACE2-positive cells and animals (Zhang et al., 2022a). By contrast, adsorption on a two-dimensional metal-based nanomaterial leads to structural alterations of the virus and its inactivation and, in addition, it promotes virus uptake and degradation by macrophages (Zhang et al., 2022b). Thus, although the interaction with biological systems occurs with the substances adsorbed on the nanoparticle surface, it is nevertheless important to assess the particle physico-chemical characteristics, because such characteristics dictate the type and amounts of adsorbed molecules and the strength of their interaction with the particle surface. Also, the binding affinity may change in different environments (e.g., in the acidic environment of lysosomes after uptake) and promote the interaction with the naked particles, or result in displacement (exchange) by proteins from the new environment with a higher surface affinity, Thus, the shed biomolecules now present in a biological compartment they do not normally reside in may affect cellular responses such as cellular proteostasis (Cai et al., 2022) potentially leading to immune response.

### 1.3 Immune response to exposure: Role of cell death and alarm signals in determining resolving vs. persistent inflammatory responses

Particle toxicity in human beings is the consequence of uptake by three main routes, inhalation, ingestion and skin contact (Alijagic et al.). The barrier function of immunity at the entry sites is mainly performed by innate immune cells, in particular phagocytic cells such as tissue-resident macrophages and, if particles induce an inflammatory reaction, neutrophils and monocytes coming from blood (Boraschi and Duschl, 2014). Along with the danger theory (Matzinger, 2002), the defensive inflammatory reaction can be initiated by exogenous PAMPs (pathogen-associated molecular patterns), or endogenous DAMPs (danger-associated molecular patterns), which activate an inflammatory reaction through innate receptors. Several types of nanoparticles, for instance crystalline silica, can kill immune cells, mostly assessed in *in vitro* models, by inducing membrane destabilisation and a chain of effects on organelle functions (Pavan and Fubini, 2017; Leinardi et al., 2022). We should remember that silica has been used by immunologists since the 1950s for macrophage depletion. The death of immune cells and in particular of the innate immune cells involved in a defensive reaction is critical to our immunity. Necrosis and immunogenic cell death (ICD), often triggered by the uptake of toxic particles by phagocytes (Galluzzi et al., 2020; Leinardi et al., 2022), are an important source of DAMPs and a source of inflammatory and immunostimulatory cytokines, such as inflammasome-generated master cytokine IL-1β. The importance of IL-1β is well established for the initiation and maintenance of inflammation, particularly upon infection (Dinarello, 2018) but also as part of the “fibre pathogenicity paradigm”: High aspect ratio particles can trigger IL-1β release from phagocytes similar to the known response to asbestos (Palomäki et al., 2011). The alarmin cytokine IL-1α, which shares the receptor with IL-1β but is inflammasome-independent, can amplify the response as shown for quartz and fibre inhalation-elicited inflammation, where IL-1α is released by lung macrophages injured upon particle uptake (Rabolli et al., 2014). On the other hand, IL-1 also facilitates the adaptive immune responses, and IL-1β is mostly an immunostimulatory factor, rather than a pure inflammatory mediator (Boraschi, 2022). Recent insights have confirmed and highlighted an important yet largely disregarded notion, i.e., that IL-1β has a role in decreasing and resolving inflammation (Giesbrecht et al., 2017) and in tissue repair (Choi et al., 2020), indicating that IL-1-initiated pathways are required for the rapid resolution of nanoparticle- (Ganguly et al., 2009), and fibre-triggered lung inflammation (Nikota et al., 2017). Persistent cell death, as observed upon inhalation of slowly cleared and highly cytotoxicity particles, such as quartz and certain rigid CNTs, will cause persistent release of alarmins and DAMPs, which will recruit more inflammatory leukocytes to the site of injury and thus eventually exacerbate the pathology (Leinardi et al., 2022). Many DAMPs have been described, mainly nuclear and mitochondrial peptides and proteins such as N-formyl peptides, histones or HMGB1, and also nuclear and mitochondrial DNA, which is sensed as DAMP if released into the cytoplasm or extracellular space, thereby serving as an injury signal. Self-DNA sensing *via* Toll-Like Receptors, inflammasomes and the cGAS/STING pathways have become a hot Research Topic for a better mechanistic understanding of lung inflammatory diseases in general (Benmerzoug et al., 2019) and particle-elicited lung inflammation in particular (Benmerzoug et al., 2018).

A recent study described immune deregulation and stress response as a shared feature across taxonomic groups (*Danio rerio, Daphnia magna* and *Chironomus riparius*) exposed to lithium cobalt oxide nanomaterials, revealing species-specific responses to the nanomaterials associated with sensitivity, both in the number and types of differentially expressed genes, and in the pathways impacted with expression (Curtis et al., 2022). There is a clear conservation of the cytokine network throughout vertebrates, with many of the key cytokines important for inflammation, innate and adaptive immunity present from fish to mammals (Zou and Secombes, 2016). However, in many cases, the genes are not true homologues and may be related to ancestral genes that have diverged independently in different vertebrate lineages. Similarly, most eukaryotic transcription factors are members of ancient protein families, and many are conserved across divergent evolutionary lineages, with the exception of the C2H2 zinc finger (ZNF) family for which, at several points in evolutionary history, novel gene types have arisen to encode proteins in which DNA-binding ZNF motifs are tethered to different types of chromatin-interacting or “effector” domains (see Liu et al., 2014). Thus, it has recently been hypothesized that C2H2-ZNF could mediate nanomaterials-induced transcriptomic responses in other species of eco-toxicological interest (del Giudice et al., 2022) including plants, where resistance protein-mediated activation of defence is based on an “altered-self” mechanism of recognition (Sanabria et al., 2010). Analysis of seventeen datasets of nanomaterials exposures to *D. rerio, Caenorhabditis elegans, Enchitraeus albidus* and *Arabidopsis thaliana* indicated that also in non-mammal organisms the adaptation response to nanomaterials is regulated by the C2H2-ZNF transcription factors family, with the relative proportion of C2H2-ZNF members decreasing down the phylogenetic tree, suggesting a possible association with organismal complexity (del Giudice et al., 2022).

Inflammation caused by particles inhalation is generally a transient defensive reaction that declines with the elimination of the nanomaterial without causing serious damage to the organism (Ganguly et al., 2009; Chen et al., 2016). It is only in a few cases, such as when the particles persist and cannot be eliminated, that the inflammatory reaction can become chronic and have pathological consequences (Boraschi et al., 2017; Leinardi et al., 2022). The use of *in vivo* animal models or the evaluation of human exposure is therefore the most reliable ways of assessing particle toxicity despite the ethical challenges and the drive towards alternative approaches. Selection of models is however very important. For instance, mice, which walk on four legs, are not a good model for inhalation exposure in humans, who stand on two, because of the completely different orientation of the lungs and consequent difference in particle localization upon inhalation. Thus, “the best model for humans is human”, but when we need alternative models, it is important that the animal species selected shares with humans the anatomical/biological characteristics we are interested in, to avoid misleading results.

Human exposure to contaminated indoor and outdoor environments naturally includes co-exposure with other pollutants present in the same environment. Among such pollutants, many can act as PAMPs (for instance bacterial and viral components), thus particles coated with PAMPs can cause inflammation by activating innate receptors of immune cells, while naked particles may show no direct effect (Li et al., 2017). The question whether nanomaterials can affect adaptive immune responses, without acting as inflammatory agents in general, can be studied by looking at type 1 allergic reactions. Type 1 (or immediate-type) allergic responses depend on Th2 cells and are thus part of adaptive immunity. There is so far no indication that nanomaterials can be true allergens (Himly et al., 2017), however, their presence may affect the sensitization process, and the degree or duration of exposure. Uptake into and processing in antigen-presenting cells is essential for loading allergen-derived peptides into the MHC-II complex, and specific nanomaterials can affect these processes (Joubert et al., 2020; Johnson et al., 2023). Thus, co-exposure to nanomaterials and allergens may have direct effects on Th2-driven adaptive immune responses. This can be relevant, for example, in the food processing industry, where airborne allergens are known to be a health issue.

### 1.4 Exposure to airborne particles and gaseous agents

A wealth of information is available on lung inflammation and toxicity caused by airborne particles, in particular elongated mineral particles (EMPs) and, more specifically, asbestos and carbon nanotubes (Aust et al., 2011; Boyles et al., 2014; Glencross et al., 2020). Notably, EMPs have in common their shape, which may contribute to their mode of interaction with biological systems and potential toxicity. However, their different chemical composition and surface characteristics may hugely affect their toxic potential. Likewise, weathering (i.e., changes in surface reactivity due to persistence in the environment, including adsorption of environmental agents) is expected to change the EMP effects on the lung. However, more recently the interest of nanotoxicologists has focused on airborne microplastics, which are abundantly present in densely populated cities and in several other environments (Liu et al., 2019; Chen et al., 2020; Li et al., 2020). The effects of airborne microplastics (with their coating of other biotic and abiotic agents) on patients with lung diseases, who have a weaker immune defensive capacity, suggests a significant risk of exacerbating the pathological symptoms, due to both mechanical and chemical impacts on lung tissue and immune reactions (Lu et al.). The gaseous composition of air, in addition to the airborne particles, can also have a profound effect on immune functions and underlie pathological derangements. In mammals, atmospheric CO_2_ concentrations above 9% cause a number of physical and neurological effects, including hypoxia and acidosis (van der Schrier et al.). Both hypoxia and acidosis have substantial effects in particular relative on innate immunity (macrophages, neutrophils), which can result in pathological immunosuppression (Lardner, 2001; Kellum et al., 2004; Riboldi and Sica, 2016; Riemann et al., 2016; Taylor and Colgan, 2017).

**FIGURE 1:**
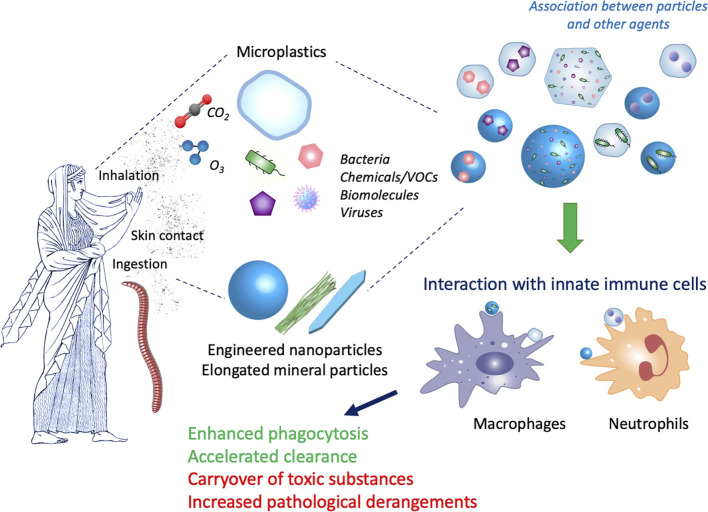
Interaction between particles and contaminants and their effect on immunity. Both vertebrates and invertebrates can be exposed to particles present in the environment, mainly by ingestion, skin contact and inhalation. Micro- and nanoparticles, e.g., microplastics, engineered nanoparticles and elongated mineral particles, are present in the environment together with many other agents, such as gases (O_3_, CO_2_), microorganisms (bacteria, viruses), chemicals (volatile organic compounds -VOCs-, synthesis residues, additives, environmental contaminants) and many different biomolecules (pollens, allergens, bacterial compounds). Depending on the particle characteristics and the environmental conditions, interaction can occur, thereby giving rise to hybrid complexes. Innate immune cells (macrophages, neutrophils) at the barrier sites (skin, lung, gut) first come in contact with the particle/contaminant complexes and react to them. Depending on the immune conditions (such as CO_2_-dependent acidosis or hypoxia, chronic diseases, immunosuppression) and the nature of the complexes, the innate immune reaction can be successful (e.g., enhanced phagocytosis and clearance of particles and microorganisms) or unsuccessful and detrimental (e.g., increased toxicity due to concentration of toxicants, increased hyperreaction/infectivity due to excessive exposure to allergens/infectious agents). Artwork by W. Yang*.*

Another important gaseous compound of urban air-pollution is the ozone (O_3_). Current regulations limit single toxicant levels but do not consider potential interactive effects of oxidant gases and nanomaterials, where both are known to develop their respiratory toxicity *via* the local generation of oxidative stress. While ozone is already well known to cause injury to the fragile alveolar region of the lungs, co-exposure can amplify single toxicant outcomes, as observed in a mouse study from Hathaway and colleagues, where progressive pulmonary mitochondrial dysfunction has been described (Hathaway et al., 2021). Eventually, volatile organic compounds (VOCs) can be released during manufacturing processes and are present in several products (paints, solvents, glues, gasoline) (Alijagic et al.). While many VOCs are known respiratory irritants, some have also shown strong toxicity and carcinogenic potential (He et al., 2015; David et al., 2021). Interaction of VOCs with nanomaterials can occur depending on the material chemical composition, surface charge, size, porosity; and nanomaterials have been used for adsorbing VOCs and also for reducing their toxicity by catalytic or photocatalytic actions (Guerra et al., 2018; Attia et al., 2019; David et al., 2021). Thus, it is might be expected that the fortuitous interaction between VOCs and nanomaterials in the environment may lead to mitigation of VOC toxicity. Figure 1 illustrates the main features of the interaction between particles and contaminants and of their effects on immunity in different types of living organisms.

## 2 Conclusion

Although the field is still in development, we can draw some general conclusions, which can be the basis for future research directions.

• Assessing conserved genes across taxa, *via* deep taxonomic comparisons, is shedding important new light on conserved immune and adaptation responses to exposure to micro/nanoscale particles, which have occurred over millennia in different forms (volcanic ash, fire and more recently manufactured and engineered materials).
• Nanotoxicity evaluation goes far beyond assessing the toxicity of particles on model cells or experimental animals, because real life exposure necessarily implies co-exposure with chemical and environmentally borne biotic and abiotic agents. Exposure studies should provide full data on bystander substances for future meta-analysis.
• Nanomaterial effects on immunity are of major importance, since anomalies in immune responsiveness may hamper homeostatic adaptation and have substantial pathological consequences.
• Combination of particles with toxic agents may have different outcomes, depending on the circumstances (nanomaterials’ chemical nature, shape, size, dose, weathering, presence of other substances, route of exposure) that will influence the mode of interaction and the overall effects on the immune responses of different living organisms.
• Endpoints of immunotoxicity should be wisely selected and longitudinally evaluated, considering that inflammation and cell death are part of a successful immune response and not necessarily linked to permanent damage or pathology.
• We must consider the possibility that interaction of particles with chemicals, biomolecules or microbes may actually abolish the potential toxicity of these substances through absorption, by altering their structure or by changing the mode of their interaction with immune cells.
• There remains a knowledge gap regarding immune reactions across living organisms generally, and a key next step in development of Adverse Outcome Pathways (AOPs), for example, could include assessing their transferability from humans to environmental species such as fish, earthworms, mussels and others widely used in ecotoxicity assessment and environmental quality monitoring.
• Although still premature, given the huge knowledge gaps we still experience, the AOP framework represents a basis for future computational investigations that do not imply the use of *in vitro* or *in vivo* experiments. It is obvious, however, that the lack of information on the individual history of previous exposure (which is not easy to assess) can substantially bias the computational results/predictions.
• A meaningful immuno-nanotoxicity evaluation requires the joint efforts and collaboration between nanotoxicologists, immunologists and epidemiologists, who should assess the history of exposure, the complexity and duration/repetition of exposures, in order to assess the longitudinal effects and predict possible pathological alterations of future immune responses.
